# Effect of Aging on Educational Differences in the Risk of Cognitive Impairment: A Gender-Specific Analysis Using Korean Longitudinal Study of Aging (2006–2016)

**DOI:** 10.3390/healthcare10061062

**Published:** 2022-06-08

**Authors:** Roeul Kim, Woojin Chung

**Affiliations:** 1 Labor Welfare Research Institute, Korea Workers’ Compensation and Welfare Service, Seoul 07254, Korea; sunset0822@hotmail.com; 2 Department of Health Policy and Management, Graduate School of Public Health, Yonsei University, Seoul 03722, Korea; 3 Institute of Health Services Research, Yonsei University, Seoul 03722, Korea

**Keywords:** cognitive impairment, education, aging, gender, longitudinal study, mixed logistic regression, Korean Longitudinal Study of Aging (KLoSA) survey, South Korea

## Abstract

This study examined the effect of aging on gender-specific educational differences in the risk of cognitive impairment using a nationally representative sample of 4278 men and 5495 women aged 45 years and older from the dataset of the Korean Longitudinal Study of Aging. Sociodemographics, lifestyle, and medical conditions were included as covariates in the mixed logistic regression analysis models. The prevalence of cognitive impairment was higher in women than in men at baseline. The risk of cognitive impairment in each age group decreased with education in both men and women. The risk by educational rank was worse at lower levels and increased with age, more so for women than men. Aging appears to widen the impact of educational differences on the risk of cognitive impairment and is more unfavorable for women than for men. Public health policies regarding population aging need to consider this and identify the target population to reduce both the level of and the difference in the risk of cognitive impairment.

## 1. Introduction

The world’s population is aging and growing at a slower pace; however, by 2050, it is still expected to increase from 7.7 billion to 9.7 billion. At that point in time, one in six individuals will be over the age of 65 (16%), up from one in 11 in 2019 (9%) [1]. This growth in the elderly population carries a variety of health challenges related to cognitive impairment, chronic diseases, and disability [2].

In particular, the increasing number of people with cognitive impairment will have a large impact on society and the healthcare system [3]. Given the lack of effective treatment, cognitive impairment will lead to deteriorating quality of life, greater risk of dementia, and higher mortality [4,5]. There were over 50 million people with dementia worldwide in 2020, and this number is projected to nearly double every 20 years, reaching 82 million in 2030 and 152 million in 2050 [6].

Significant studies have already been performed to identify and explore factors related to the risk of cognitive impairment, including age, education level, brain injury, family history, physical inactivity, and chronic diseases such as high cholesterol, peripheral vascular disease, hypertension, obesity, kidney disease, stroke, and diabetes [7,8,9]. Nevertheless, these studies appear to have been subject to various limitations, including (1) a lack of nationally representative samples [10,11,12,13,14,15,16,17,18,19,20,21]; (2) insufficiency of adult age groups [5,10,11,12,15,16,22,23], (3) absence of gender-specific analyses [11,12,13,24], (4) failure to use longitudinal data analysis methods [10,14,15], and (4) deficiencies regarding covariates [11,12,13,14,15,16,17,25,26,27].

Meanwhile, a few other studies have examined education level, gender, and age in relation to cognitive decline [16,25]. The analysis of 2347 individuals from the Doetinchem Cohort Study revealed that an unhealthy lifestyle and relatively poor health in midlife were significantly associated with worsened cognition 10 years later, regardless of gender or educational level [25]. Unfortunately, the small sample size was limited, and occupation, although important, was not assessed as a possible risk factor for cognitive impairment [28,29]. Subsequently, the testing of visuospatial working memory performance among 134 individuals between 20 and 80 years of age in Italy revealed that educational level was positively associated with working memory performance. In their study, education and age were not interactively associated. Notably, this effort was restricted by both the sample size and the exclusion of potential covariates.

There is a substantial gap in the literature regarding the relationship between education, gender, and age and the risk of cognitive impairment. To the best of our knowledge, this is the first study to address this deficiency by examining how the impact of educational differences on this risk changes with age and gender. We analyzed six waves of data from a nationally representative longitudinal survey in South Korea (hereafter, Korea) using a mixed logistic regression analysis involving three statistical models. A growth curve analysis was applied to determine the probability of cognitive impairment during the aging of a given man or woman, ranked by level of education.

## 2. Materials and Methods

### 2.1. Data Source and Study Sample

This study is part of a broad-ranged study exploring the characteristics associated with the risk of cognitive impairment in South Korea, which includes a series of different studies we had previously carried out [28,29,30]. Therefore, although the present study is completely different from previous studies in terms of specific research topics, we want to report that the studies have similarities, to some extent, in study materials and methods. Instead of omitting repeated parts of the study materials and methods from previous studies, we will provide their detailed descriptions in the current study for the readers’ convenience.

The Korean Longitudinal Study of Aging (KLoSA) focused on Koreans aged 45 years and older living in households selected by multistage stratified probability sampling to be representative of the nation. A total of 10,254 participants completed the interviews conducted by trained interviewers during the baseline survey. This study employed the KLoSA data from 2006 to 2016. Detailed information regarding the survey is available on the KLoSA website (https://survey.keis.or.kr/klosa/klosa01.jsp, accessed on 20 July 2021).

We restricted the participants to those surveyed both at baseline in 2006 (i.e., the first wave) and at least once in later assessments. Of the resulting 47,995 observations, those excluded were enumerated in the following categories: (1) non-contact, refusal, or death (1474); (2) intellectual disability diagnoses, organic brain diseases, and/or psychiatric treatments (1777); and (3) non-reporting of the Korean Mini-Mental State Examination (K-MMSE) score (1707). The final study sample comprised 9773 participants of the first wave and 43,037 total observations, with an average of 4.33 observations per participant (standard deviation, 1.99; range, 1–6). As for the number of observations that participants contributed to, 5002 participants contributed six times, 957 participants contributed five times, 616 participants contributed four times, 712 participants contributed three times, 995 participants contributed twice, and 1650 participants contributed once. Informed consent was obtained from all participants when the KLoSA survey was conducted, in accordance with the ethical principles of the Declaration of Helsinki. This study was approved by the institutional review board of thee Severance Hospital (Y-2018-0138).

### 2.2. Measures and Variables

Cognitive function was assessed using the K-MMSE [31,32]. The K-MMSE assesses orientation, recall, language, registration, attention, calculation, and the ability to follow simple commands with a sensitivity of 0.70–0.83 in detecting dementia. The total score ranges from 0 to 30, with higher scores indicating better cognitive function. Cognitive impairment was defined as a K-MMSE score of less than 24 [33,34]. Therefore, a dichotomous outcome variable was constructed with values of 1 (cognitive impairment; K-MMSE score < 24) and 0 (no cognitive impairment; K-MMSE score ≥ 24).

Age, gender, and education level were selected as variables of interest. For descriptive and univariate analyses, we categorized age into four groups (45–64, 65–74, 75–84, and ≥85 years); however, for multivariable analyses, we used a centered age (age minus its mean value) and its squared value to reduce potential multicollinearity. The education levels of the respondents were categorized as elementary school or lower, middle school, high school, and college or higher.

We included multiple covariates in the analysis, including socioeconomic status, psychosocial and behavioral factors, and health status. Socioeconomic status included household income, occupation (white-collar, blue-collar, or no job), and home ownership. Household income in each wave was adjusted for household size using the square root equivalence scale [35] and divided into three groups: two groups based on the median value and, to avoid losing valuable information, a third group of participants who did not report their household income. Psychosocial factors included marital status (married or non-married, where non-married included never-married, separated, widowed, and divorced), residential area (urban or rural), religion (yes or no), and depressive symptoms (yes or no). Depressive symptoms were assigned scores of four or more on the 10-item short form of the Center for Epidemiologic Studies Depression Scale (CES-D10) [36,37]. Behavioral factors included drinking (yes or no), smoking (yes or no), and routine physical exercise (yes or no). Routine physical exercise was assessed by asking the participants whether they engaged in any physical exercise at least once a week for their own health. Health-related factors included obesity (yes or no) and chronic disease (yes or no). We described obesity as a body mass index of at least 25 based on the revised Asia-Pacific criteria by the World Health Organization of the Western Pacific Region [38]. Chronic disease was assessed from self-reported answers to survey questions about the medical history of clinical diagnosis of hypertension, diabetes, cancer, chronic lung disease, chronic hepatitis, cerebrovascular diseases, mental diseases, or arthritis.

### 2.3. Statistical Analysis

First, three statistical models were established. Model 1 was a cross-sectional model, where, for participants at baseline (Wave 1), we estimated the prevalence rate of cognitive impairment across age groups and education levels by gender. Models 2 and 3 were longitudinal models for all observations of all considered waves. Models 2 and 3 used a dichotomous outcome variable for cognitive impairment and included age, gender, and education level as variables of interest. Model 2 did not include any covariates, but Model 3 included the full set of covariates, such as socioeconomic status, psychosocial and behavioral factors, and health status. In Models 2 and 3, we employed mixed logistic regression with two levels, because longitudinal dataset observations are likely to be temporally correlated within the same participant. Furthermore, we attempted to avoid potential bias in parameter estimates for multilevel logistic regression models with small samples by scaling the conditional weights at level 1 of the data hierarchy and normalizing them to an intra-cluster sample size [39,40].

Before embarking on a detailed analysis, we needed to decide whether all analyses should be stratified by gender. To do this, we tested the following null hypotheses: (1) the risk of cognitive impairment is the same between genders, (2) the association between age and cognitive impairment is the same between genders, and (3) the association between education and cognitive impairment is the same between genders. The evaluation involved applying the chi-square and Wald tests, each with both main-effect terms and an interaction-effect term, in approaches that were logistic for Model 1 and linear for Models 2 and 3. Consequently, we rejected the hypothesis that the risk of cognitive impairment is the same between genders in Models 1 to 3 (*p* < 0.0001). As for the hypothesis that the association between age and cognitive impairment is the same between genders, we rejected it absolutely in Model 1 (*p* < 0.0001) and marginally in Model 3 (*p* = 0.0509), but we could not reject it in Model 2 (*p* = 0.1180). Moreover, we rejected the hypothesis that the association between education level and cognitive impairment is the same between genders in Models 1 and 3 (*p* < 0.01). Based on these results, we stratified all analyses by gender.

For multivariable analyses, we used several steps to determine the appropriate model specifications. First, we continued to reclassify each variable and redefined its reference category. As a result, throughout all models, the values of the variance inflation factor became <2.26, implying no strong multicollinearity, and the *p*-values based on the Hosmer–Lemeshow statistic became >0.970, demonstrating no evidence of a lack of goodness-of-fit. Subsequently, using the pseudo Akaike information criterion as a measure of the goodness-of-fit of the mixed model, we selected a random intercept model along with an unstructured diagonal covariance structure. All covariance parameter estimates for each model were significant (*p* < 0.0001), suggesting that each model was adjusted for a considerable degree of correlation between the observations within an individual. The null model revealed a high degree of intraclass correlation (0.675 and standard error 0.015 for men and 0.758 and standard error 0.010 for women).

We then investigated how the risk of cognitive impairment in participants with a particular educational level changed during aging. To do this, using the results of Model 3, we evaluated changes in an individual’s predicted probability of having cognitive impairment (and its 95% confidence interval [CI]) between 45 and 90 years of age for each gender and education level using a delta method and then drew growth curves. Each predicted probability of having cognitive impairment could be interpreted as a predicted value of the probability of having cognitive impairment that a participant with a particular education level would have at a particular age; all the other characteristics were maintained as they were at the participant’s values.

Throughout every estimation process, we considered all characteristics as time-dependent (susceptible to change as time progressed) and estimated the odds ratios (ORs) and 95% CIs. Statistical significance was set at *p* < 0.05 (two-tailed) for statistical significance. SAS software (version 9.4; SAS Institute, Cary, NC, USA) and STATA 15 software (StataCorp., College Station, TX, USA) were used to perform all statistical analyses.

## 3. Results

For comparison of the characteristics of the sample participants at baseline (Wave 1) by gender, the mean cognitive function score was higher in men than in women (26.7 vs. 24.6), but women were older on average than men (61.8 vs. 61.1 years) (Table 1). Compared to men, women had a higher proportion in each of the following categories: aged 75–84 years, aged 85 years and above, non-married, religious, residing in an urban area, attaining an educational level of elementary school or less, having no job, belonging to the lower-half group of household income, belonging to the group of individuals who did not report household income, house renter, non-smoking, non-alcohol intake, inactive routine physical exercise, non-obese, having a chronic disease, or having depressive symptoms.

According to the results from Model 1, the prevalence of cognitive impairment was almost three times higher in women (26.4%, 95% CI: 25.3–27.6%) than in men (10.9%, 95% CI: 10.0–11.9%), showing a significant difference between genders (Rao-Scott Chi-square test, *p* < 0.0001) (Table 2). The prevalence rate differed across age categories (Rao-Scott Chi-square test, *p* < 0.0001) and had a positive linear trend with aging (Wald test, *p* < 0.0001) for each gender. The prevalence increased sharply from participants aged 45–64 years to participants aged 85 years and above for each gender, from 5.5% to 64.6% in men and from 11.5% to 88.7% in women. Therefore, according to the results of Model 1, the risk of cognitive impairment varied with age.

The prevalence of cognitive impairment varied across education levels (Rao-Scott Chi-square test, *p* < 0.0001), showing a negative, linear trend with higher education level for each gender (Wald test, *p* < 0.0001). For each sex, the prevalence rate fell from participants with an education level of elementary school or less (26.4% in men and 46.9% in women) to participants with an education level of college or higher (2.4% in men and 1.6% in women). Therefore, in Model 1, the risk of cognitive impairment differed across educational levels.

Regarding the results from Model 2 (the first and second of the four OR columns in Table 3), the risk of cognitive impairment for each gender increased rapidly with age but decreased with education level. Therefore, the risk of cognitive impairment varied with age in Model 2 (Wald test, *p* < 0.0001) and that the risk of cognitive impairment differed across education levels (Wald test, *p* < 0.0001).

Concerning the results of Model 3 (the third and fourth of the four OR columns in Table 3), the risk of cognitive impairment in men increased rapidly with age. Relative to their respective mean values, the ORs of cognitive impairment for age and age squared were 1.08 (95% CI, 1.06–1.09) and 1.00 (95% CI, 1.00–1.00), respectively. Note that the difference in the OR between age squared and its mean value was very small but significantly positive. Meanwhile, the risk of cognitive impairment decreased with education level. Individuals with a college or higher level of education had an OR of cognitive impairment of 0.16 (95% CI, 0.12–0.22) in comparison with individuals with elementary school or lower level of education.

Similar patterns were observed in women, except that the OR of cognitive impairment for age was 1.11 (95% CI, 1.10–1.12) (slightly higher than for men), and the OR for participants with college or higher level of education was 0.08 (95% CI, 0.04–0.13) (lower than for men).

Therefore, in both men and women, Model 3 revealed that the risk of cognitive impairment varied with age (Wald test, *p* < 0.0001) and across education levels (Wald test, *p* < 0.0001). Apart from a few covariates, most were found to be statistically significant, with the exceptions being marital status, residential area, home ownership, smoking and obesity in men, and smoking and chronic disease in women.

**Figure 1:**
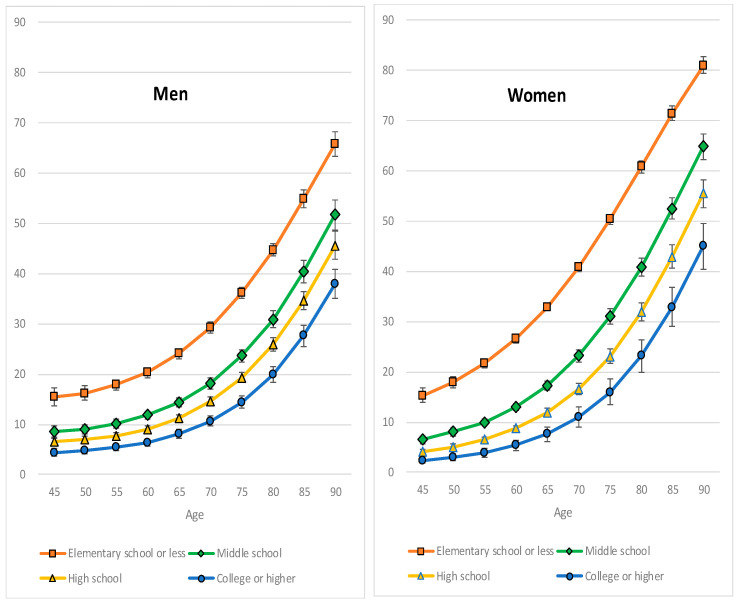
Gender-specific changes in the predicted probability (%) of having a cognitive impairment for each education level and their 95% confidence intervals with aging for all observations of all considered waves.

Irrespective of gender, one’s predicted probability (%) of having cognitive impairment decreases with education level but increases with age (Figure 1). However, the growth curve, which shows a positive relationship between the predicted probability of cognitive impairment and aging, was steeper for a lower level of education than for a higher level of education, and it was also steeper in women than in men. For example, in men, the predicted probabilities of having cognitive impairment for the elementary school or less category and the college or higher category at the age of 45 years were 15.4% (95% CI, 11.8–19.0%) and 4.4% (95% CI, 3.0–5.9%), respectively. At the age of 90 years, the probabilities were 65.8% (95% CI, 61.9–70.4%) and 37.9% (95% CI, 32.1–43.6%), respectively. In women, at the age of 45 years, the values were 15.3% (95% CI, 12.5–18.1%) and 2.4% (95% CI, 1.3–3.5%), respectively, whereas at the age of 90 years, probabilities of 81.0% (95% CI, 77.8–84.2%) and 45.0% (95% CI, 36.0–54.1%) were observed, respectively. Meanwhile, the differences in the predicted probability of having cognitive impairment between the elementary school or less category and the college or higher category became larger during aging for each gender. These differences at each age were greater in women than in men: 11.0 percentage points at the age of 45 years and 27.9 percentage points at the age of 90 years in men, and 12.9 percentage points at the age of 45 years and 36.0 percentage points at the age of 90 years in women.

## 4. Discussion

Prior research has found that women are more likely than men to suffer from cognitive impairment, which is in line with our findings [17,26,41,42]. An analysis of 34,439 cognitively normal elderly individuals in five North American prospective cohort studies from 1971 to 2017 revealed that women might have greater cognitive reserve but faster cognitive decline than men, which could contribute to gender differences in late-life dementia [17]. Specifically, compared with men, women had significantly faster declines in both global cognition and executive function, but not in memory. Furthermore, tracking with repeated measures of cognition in a 15-year French cohort of 2228 adults older than 65 years of age (PAGUID) suggested that women had a slightly steeper global cognitive decline with aging than men after adjusting for age, education, and vascular diseases [26]. The reasons for gender differences are complex and likely influenced by genetic (APOE), biological (sex hormones), social, and cultural factors [43].

Nevertheless, data from the longitudinal Berlin Aging Study did not show any relationship between gender and the degree of cognitive decline with aging [18]. Likewise, no gender-based differences in semantic and episodic memory changes over time were revealed when data from the population registry in Umea (Northern Sweden) were analyzed [19]. Notably, both studies were limited by the relatively small sample sizes (368 and 361 participants, respectively) and the lack of inclusion of different potential covariates.

Many previous studies have indicated that the risk of cognitive impairment at each age declines with education level in both men and women [20,24]. The community-based Washington Heights-Inwood Columbia Aging Project generated data showing that more years of education were associated with higher cognitive levels and slower cognitive decline with aging [20]. Cognitive decline was observed in all age groups of MMSE-qualified participants (1488) of the Baltimore cohort of the Epidemiological Catchment Area study, and having more than eight years of formal education was associated with less cognitive decline in both men and women [24].

A Canberra longitudinal study of 887 Australians aged 70–93 years employed latent growth curve models and more traditional regression to show that education was not associated with changes in global cognition, memory, cognitive speed, or crystallized intelligence [21]. Data from 1014 participants aged 54–95 years at baseline in the Victoria Longitudinal Study in Canada demonstrated that education was related to cognitive performance but unrelated to cognitive decline [27]. The findings support the passive cognitive reserve hypothesis, in which individuals with greater educational attainment continue to perform at a higher level than similarly aged individuals with less education but decline at a similar rate [44]. One limitation in this instance was the very small size of the subset of individuals with less than 9 years of education (3%).

In the present study, we found that the risk of cognitive impairment at each education level increased with age and that its worsening with age was more severe in women than in men and at lower levels of education.

Previous studies found that, in both men and women, the risk of cognitive impairment decreased with education level but increased with age [22,45,46]. A study of 659 cognitively normal elders who completed neuropsychological tests demonstrated that higher education in early life was a protective factor in aging, which may help postpone brain reserve capacity decline with cognitively normal aging [46]. Likewise, a study using data from the National Alzheimer’s Coordinating Center Minimum and Neuropathology Data Sets found that highly educated elders with neuropathological Alzheimer’s disease were less likely to have a dementia diagnosis than their counterparts [22]. These results support the theory that persons with greater cognitive reserve, as reflected in years of education, are able to withstand with Alzheimer’s disease pathology without observable cognitive decline.

Overall, elderly people who are more educated appear to be less susceptible to age-related and pathological cognitive changes. However, the mechanism underlying the protective effects of education on cognitive aging remains unclear. One possible explanation is that education protects cognition through life activities from the perspective of environmental factors [46]. In keeping with the use-it-or-lose-it hypothesis, many studies have revealed that mental stimulation in early life protects cognitive function in older age [29,47]. Another possible explanation is that educational attainment is associated with brain reserves. Higher numbers of years of education have been related to improvements with age in several brain MRI indices, including greater cerebral [46] or gray matter volume [48] and more favorable white matter macrostructure and microstructure [49].

Our finding of greater educational impact with increased age on the risk of cognitive impairment is consistent with ideas supported by various analyses. As others have suggested [24,45,50,51], it is possible that educational attainment moderates the trajectory of normal age-related cognitive decline. For example, a representative sample of 70-year-old Americans from four waves of the Asset and Health Dynamics Among the Oldest Old study (AHEAD) showed that higher educational attainment was related to better initial performance on cognitive tests and that higher levels of education tended to slow the decline in mental status [50]. This slowing of decline in general mental status supports an active cognitive reserve hypothesis, such that persons with higher educational attainment may process tasks more efficiently. Because these individuals make more efficient use of brain networks, the same amount of organic cognitive damage results in a smaller reduction in their cognitive function than that in those with less education.

Not to be overlooked, there are reports that the effect of education on age-related decline is restricted to specific cognitive domains [42,52]. A study analyzing harmonized longitudinal data for 14 cohorts from 12 countries (Australia, Brazil, France, Greece, Hong Kong, Italy, Japan, Singapore, Spain, Korea, the UK, and the USA) with a total of 42,170 persons aged 54–105 years found a negative association between years of education and the rate of decline in the Mini-Mental State Examination (MMSE) [42].

This study also found that as women aged, they were more likely than men to be exposed to the risk of cognitive impairment, irrespective of their level of education. These gender-based differences conform with those described in previous studies [26,42]. They may be influenced in complex ways by the effects of longevity (women live longer than men) [53], biological differences (hormonal differences, epigenetics, and frailty) [54,55], and gendered social roles and opportunities (educational and occupational opportunities, and post-retirement functional roles) [56]. One interesting theory is that gender may influence the clinical diagnosis of cognitive impairment and dementia. For instance, after retirement, women are more likely than men to engage in diverse household chores. Declines in functional abilities, which are key to making a diagnosis of cognitive impairment, would therefore be more readily detected by family and friends in elderly women than in now-retired men whose roles typically involved working at outside jobs without doing much in the house [56]. Women are more often engaged in family caregiving activities, and caregiving itself is associated with an increased risk of cognitive impairment and dementia [28,29,57,58].

Concerning implications for public health, the findings of the present study argue for policies that lessen the effect of educational differences on the risk of cognitive impairment both by gender and across genders, since these respective differences increase with age. The highest priority should be women with the lowest levels of education, because they are exposed to the highest risk of cognitive impairment. Policies should favor preventive measures for those who are younger or middle-aged, while emphasizing treatment and alleviation for older people.

Furthermore, considering that education is reported to be one of the best-established preventive measures for cognitive impairment [59], young people in general should be encouraged to attain higher levels of education as much as possible. Data from 15,924 persons born between 1930 and 1955 in the UK and enrolled in two prospective cohort studies were analyzed to examine the role of education in influencing gender differences in cognitive aging. After accounting for the level of education, they revealed no evidence of cognitive disadvantage in women. The implication is that decreasing educational disparities between genders by improving opportunities for learning could attenuate gender differences in cognitive decline in the future [60].

For elderly people experiencing cognitive decline who have already completed their formal and occupational education [61], it is worth considering that encouragement to engage in a diversity of social activities may be very helpful. Previous studies suggest that significant associations exist between cognitively activating leisure activities, engagement in social activities, and level of cognitive performance and the risk of dementia [62,63]. There is also evidence that cognitively stimulating leisure activities later in life offer a degree of compensation for low educational attainment [64,65].

Significant advances in this area of research require an appreciation of its multifactorial nature and the rigorous application of appropriate methodology. Although we have made progress with this particular effort, it is important to recognize the distinctive features that relate to advantages and disadvantages. Notably, ours is the first study to investigate the effects of age and gender on educational differences in the risk of cognitive impairment using a nationally representative longitudinal dataset and time-varying covariates in a mixed model analysis. In-depth, three-dimensional analyses with a cross-sectional analysis, a longitudinal analysis with no covariates, and a longitudinal analysis with all studied covariates were components of this investigation.

There are a few limitations to this study. First, there were 1474 missing observations due to non-contact, refusal, or death; 1177 missing observations due to diagnosis of intellectual disability, organic brain disease, or treatment for psychiatric illness; and 1707 missing observations due to non-reporting of the K-MMSE scores. Second, cognitive function was measured using the K-MMSE on the basis of respondents’ self-reports, without other clinical assessments, such as a clinical dementia rating scale or neuropsychological battery [66]. Thus, it may not reflect actual capabilities or adequately address cognitive functional problems. However, the MMSE is a convenient alternative measure for detailed neuropsychological testing [67]. Its use allowed us to evaluate cognitive changes in a large number of subjects [66,68]. Future studies should use more comprehensive neuropsychological or other cognitive indices to evaluate cognitive function. Third, we used binary variables for depressive symptoms from the Center for Epidemiologic Studies Depression Scale (CES-D10). Depressive symptoms were assigned scores of four or more on the 10-item short form of the CES-D10 [36,37] in this study. However, the CES-D10 has not been validated using any gold-standard clinical measure [69]. Future studies should consider a more detailed depression test to evaluate cognitive functions, since depression is often associated with cognitive problems [70]. Fourth, ascertainment of lifestyle and health factors was based only on self-reports of smoking, alcohol intake, and chronic disease, which may be susceptible to response bias. Finally, regarding the risk of cognitive impairment, we chose to consider educational differences rather than income differences because education level is usually determined earlier in life (with a likely continuing effect on income level) and because current cognitive impairment may affect current income level, thereby fostering a reverse causality problem.

## 5. Conclusions

This study provides evidence of the effect of aging on educational differences in the risk of cognitive impairment by gender in middle-aged and older adults using a dataset from a Korean national longitudinal study. We found that, while rising with increasing age for both men and women, the risk of cognitive impairment at each age decreased as the level of education increased. Furthermore, irrespective of gender, the effect of educational difference on the risk of cognitive impairment worsens with age, and it worsens with age at a greater rate in women than in men. Further research is needed to investigate whether these results and corresponding suggestions are valid in other settings, in terms of sociocultural or economic development.

## Figures and Tables

**Table 1:** The characteristics of sample participants by gender at the baseline (Wave 1).

Characteristics	Men	Women
Cognitive function score: Mean (SD)	26.7 (4.2)	24.6 (5.7)
Age, years: Mean (SD)	61.1 (10.5)	61.8 (11.4)
45–64	61.2%	59.1%
65–75	27.2%	25.2%
75–84	10.2%	13.3%
85 and above	1.4%	2.4%
Non-married	7.8%	32.4%
Religion, yes	44.5%	63.9%
Resides in a rural area	22.9%	22.8%
Education level		
Elementary school or less	31.6%	58.2%
Middle school	17.0%	15.6%
High school	33.8%	21.3%
College or higher	17.6%	4.9%
Occupation		
No job	43.3%	76.0%
Blue collar job	40.6%	20.5%
White collar job	16.1%	3.5%
Household income		
Lower half	44.0%	47.6%
Higher half	49.1%	43.6%
Unreported	6.9%	8.8%
House renter	21.3%	24.2%
Smoking, yes	40.6%	3.1%
Alcohol intake, yes	64.0%	18.7%
Routine physical exercise, active	43.1%	35.3%
Obese, yes	21.3%	23.1%
Chronic disease, yes	37.8%	39.1%
Depressive symptoms, yes	24.1%	35.4%
Number of observations	4278	5495

Note: SD denotes standard deviation.

**Table 2:** Prevalence of cognitive impairment across age groups and education levels by gender at the baseline (Wave 1) and the distribution of observations across age groups and education levels by wave.

Characteristics	Prevalence (%)				Distribution of Observations (%)						
	Men		Women								
	Rate	(95% CI)	Rate	(95% CI)	Wave 1	Wave 2	Wave 3	Wave 4	Wave 5	Wave 6	Overall
Overall	10.9	(10.0–11.9)	26.4	(25.3–27.6)							
Chi-square test, *p*-value			<0.0001								
Age, years											
45–64	5.7	(4.8–6.6)	11.5	(10.5–12.7)	60.0	54.3	50.0	45.0	40.4	31.4	48.6
65–75	21.6	(19.2–24.3)	47.3	(44.5–50.1)	26.1	28.6	29.8	30.8	31.4	32.8	29.5
75–84	40.2	(35.4–45.1)	76.9	(73.6–79.9)	12.0	14.2	16.7	19.9	22.8	27.4	17.9
85 and above	64.6	(50.4–76.7)	88.7	(79.8–94.0)	1.9	2.9	3.5	4.3	5.4	8.4	4.0
Chi-square test, *p*-value		<0.0001		<0.0001							
Linear trend test, *p*-value		<0.0001		<0.0001							
Education level											
Elementary school or less	26.4	(24.0–28.9)	46.9	(45.0–48.7)	46.6	46.9	46.8	46.1	44.9	43.6	46.0
Middle school	9.9	(7.8–12.3)	10.3	(8.4–12.7)	16.2	16.2	16.7	16.9	17.1	17.3	16.6
High school	6.0	(4.8–7.4)	3.5	(2.6–4.7)	26.8	27.0	26.9	27.2	27.9	30.1	27.5
College or higher	2.4	(1.5–3.7)	1.6	(0.6–3.8)	10.4	9.9	9.6	9.8	10.1	9.0	9.9
Chi-squared test, *p*-value		<0.0001		<0.0001							
Linear trend test, *p*-value		<0.0001		<0.0001							
Number of observations	4278		5495		9773	8131	7111	6503	5996	5523	43,037

Note: CI denotes confidence interval. Prevalence estimation and tests were performed by considering a complex sampling design.

**Table 3:** Associations of age and education with cognitive impairment by gender for all observations of all considered waves.

Characteristics	Model with No Covariate						Model with All Studied Covariates					
	Men			Women			Men			Women		
	OR	(95% CI)	*p*	OR	(95% CI)	*p*	OR	(95% CI)	*p*	OR	(95% CI)	*p*
Age	1.11	(1.10–1.13)	<0.001	1.13	(1.12–1.14)	<0.001	1.08	(1.06–1.09)	<0.001	1.11	(1.10–0.12)	<0.001
Age squared	1.00	(1.00–1.00)	<0.001	1.00	(1.00–1.00)	<0.001	1.00	(1.00–1.00)	<0.001	1.00	(1.00–1.00)	<0.001
Education level (Ref: Elementary school or less)												
Middle school	0.35	(0.27–0.44)	<0.001	0.21	(0.17–0.26)	<0.001	0.40	(0.32–0.50)	<0.001	0.27	(0.23–0.34)	<0.001
High school	0.20	(0.16–0.25)	<0.001	0.10	(0.08–0.12)	<0.001	0.27	(0.22–0.34)	<0.001	0.15	(0.12–0.18)	<0.001
College or higher	0.11	(0.08–0.15)	<0.001	0.04	(0.03–0.08)	<0.001	0.16	(0.12–0.22)	<0.001	0.08	(0.04–0.13)	<0.001
Non-married (Ref: Married)							1.25	(0.98–1.59)	0.069	1.28	(1.11–1.48)	0.001
Religion (Ref: No)							0.86	(0.75–0.99)	0.042	0.75	(0.67–0.84)	<0.001
Resides in a rural area (Ref: Reside in a urban area)							0.94	(0.78–1.13)	0.503	1.49	(1.27–1.73)	<0.001
Occupation (Ref: No job)												
Blue collar job							0.49	(0.41–0.58)	<0.001	0.61	(0.53–0.70)	<0.001
White collar job							0.48	(0.35–0.66)	<0.001	0.27	(0.15–0.49)	<0.001
Household income, higher half (Ref: Lower half and unreported)							0.81	(0.70–0.94)	0.004	0.78	(0.70–0.87)	<0.001
House renter (Ref: House owner)							1.07	(0.88–1.31)	0.493	1.32	(1.13–1.54)	<0.001
Smoking, yes (Ref: Non-smoking)							0.90	(0.76–1.06)	0.202	1.12	(0.78–1.62)	0.545
Alcohol intake, yes (Ref: Non-alcohol intake)							0.81	(0.69–0.94)	0.005	0.79	(0.66–0.94)	0.007
Active physical exercise (Ref: Inactive)							0.53	(0.46–0.61)	<0.001	0.63	(0.56–0.71)	<0.001
Obese, yes (Ref: Non-obese)							0.86	(0.71–1.04)	0.111	0.80	(0.70–0.92)	0.002
Chronic disease, yes (Ref: No)							1.24	(1.07–1.45)	0.005	1.12	(0.99–1.28)	0.071
Depressive symptom, yes (Ref: No)							2.20	(1.94–2.49)	<0.001	1.98	(1.79–2.18)	<0.001
Number of observations	18,654			24,383			18,654			24,383		

Note: OR denotes odds ratio. CI denotes confidence interval. Age was centered around its mean. Non-married included never-married, separated, widowed, and/or divorced. Household income was adjusted for household size for each wave. Obesity was defined as a body mass index ≥25. Depressive symptoms were defined as a score ≥4 on the 10-item short form of the Center for Epidemiologic Studies Depression Scale. The effects of the continuous variables, age and age squared, were assessed as one unit offset from its mean. All values were estimated using a complex sampling design. All characteristics were considered time-dependent.

## Data Availability

Data from authors will be available upon request.
